# *MC1R* variants and associations with pigmentation characteristics and genetic ancestry in a Hispanic, predominately Puerto Rican, population

**DOI:** 10.1038/s41598-020-64019-y

**Published:** 2020-04-29

**Authors:** Amelia K. Smit, Marielys Collazo-Roman, Susan T. Vadaparampil, Stella Valavanis, Jocelyn Del Rio, Brenda Soto, Idhaliz Flores, Julie Dutil, Peter A. Kanetsky

**Affiliations:** 1 Cancer Epidemiology and Prevention Research, Sydney School of Public Health, Faculty of Medicine and Health, The University of Sydney, Sydney, Australia; 2 Melanoma Institute Australia (MIA), The University of Sydney, Sydney, Australia; 3 School of Medicine, Ponce Health Science University, Ponce, Puerto Rico; 4 Department of Health Outcomes and Behavior, Moffitt Cancer Center and Research Institute, Tampa, FL USA; 5 Department of Cancer Epidemiology, Moffitt Cancer Center and Research Institute, Tampa, FL USA; 6 Public Health Program, Ponce Health Science University, Ponce, Puerto Rico; 7 Cancer Biology Division, Ponce Research Institute, Ponce Health Science University, Ponce, Puerto Rico

**Keywords:** Genetics research, Disease prevention

## Abstract

Skin cancer risk information based on melanocortin-1 receptor (*MC1R*) variants could inform prevention and screening recommendations for Hispanics, but limited evidence exists on the impact of *MC1R* variants in Hispanic populations. We studied Hispanic subjects, predominately of Puerto Rican heritage, from Tampa, Florida, US, and Ponce, PR. Blood or saliva samples were collected by prospective recruitment or retrieved from biobanks for genotyping of *MC1R* variants and ancestry informative markers. Participant demographic and self-reported phenotypic information was collected via biobank records or questionnaires. We determined associations of *MC1R* genetic risk categories and phenotypic variables and genetic ancestry. Over half of participants carried *MC1R* variants known to increase risk of skin cancer, and there was diversity in the observed variants across sample populations. Associations between *MC1R* genetic risk groups and some pigmentation characteristics were identified. Among Puerto Ricans, the proportion of participants carrying *MC1R* variants imparting elevated skin cancer risk was consistent across quartiles of European, African, and Native American genetic ancestry. These findings demonstrate that *MC1R* variants are important for pigmentation characteristics in Hispanics and that carriage of high risk *MC1R* alleles occurs even among Hispanics with stronger African or Native American genetic ancestry.

## Introduction

As knowledge of associations between common genetic variants and elevated disease risk rapidly advances, expectations are high for the implementation of genetic testing in routine clinical practice and public health strategies^1–4^. For preventable cancers such as skin cancer, identification of common genetic variants can improve the precision of risk prediction models and inform risk-stratified prevention and early detection strategies to potentially reduce mortality, morbidity and healthcare costs^5^.

Numerous studies have demonstrated that inherited genetic variation in the melanocortin-1 receptor (*MC1R*) gene, a primary regulator of skin pigmentation, is associated with increased risk of melanoma and non-melanoma (keratinocyte) skin cancers, including basal cell and squamous cell carcinomas^6–8^. Among individuals with ‘sun-resistant’ phenotypes (e.g. good tanning response, dark hair, low burnability), associations between *MC1R* variants and skin cancer are often stronger than those among individuals with ‘sun-sensitive’ phenotypes (e.g. poor tanning response, red or blond hair, burnable skin)^8,9^. It is likely that individuals with sun resistant phenotypes who carry *MC1R* risk genotypes may be unaware of their elevated skin cancer risk. However, most genetic epidemiology studies of skin cancer to date have focused on non-Hispanic white populations, which potentially limits the generalizability of skin cancer risk information based on *MC1R* genotypes to at-risk populations with more diverse genetic ancestry, such as Hispanics.

The incidence of melanoma—the deadliest form of skin cancer—is rising among Hispanics^10,11^. Although Hispanics have a lower lifetime risk of melanoma than non-Hispanics, Hispanics are more likely to be diagnosed at a younger age, have higher disease morbidity, and experience late stage clinical presentation of melanoma leading to higher mortality rates^11–14^. Alongside increasing melanoma incidence, over 6000 new cases of non-melanoma skin cancers were diagnosed among Hispanics living in Puerto Rico in 2005, which represents about a 300% increase since 1974^15^. In addition to well-established factors such as unequal access to healthcare that influence disparities in health outcomes for Hispanics, poorer outcomes may also be impacted by public health efforts focusing on sun-sensitive phenotypes and a lack of patient and clinician awareness about skin cancer risk in Hispanics^16,17^. Prevention and screening advice that incorporates *MC1R* genotypes may improve skin cancer risk awareness and risk reduction among Hispanics^18^, but evidence on *MC1R* variants and their associations with pigmentation characteristics in Hispanic populations is limited.

As a prelude to conducting an intervention study among Hispanics to determine whether feedback of *MC1R* genotype (i.e. precision prevention) can affect change in skin cancer prevention behaviors, we first addressed some gaps in research evidence by conducting a pilot study to examine the prevalence of *MC1R* variants among Hispanics living in the Tampa Bay region of Florida, US and in Puerto Rico. These geographies were selected because of an ongoing federally-funded partnership initiative between Ponce Health Sciences University (PHSU) in Ponce, PR, and Moffitt Cancer Center (MCC) in Tampa, Florida, US, the overall goal of which is to improve cancer care outcomes for Hispanics in Puerto Rico and Florida. We further assessed associations between *MC1R* variation and traditional skin cancer risk factors and genetic ancestry in this population.

## Materials and methods

### Subjects and data collection

Three different sources were used to obtain samples and/or recruit study participants: the Puerto Rico Biobank (PRBB), located in Ponce, PR; a Community Participant Registry (CPR) covering Puerto Rico and Florida, US; and the Morsani Family Medicine clinics (MFMC) at the University of South Florida, in Tampa, Florida, US. All participant information, informed consent forms, and questionnaires were available in both Spanish and English. We also obtained genotype data from the 1000 Genomes project.

#### Puerto Rico Biobank (PRBB)

The PRBB is a cancer tissue biobank housed at PHSU that was established as part of a PHSU-MCC partnership initiative. In order to contribute to the PRBB, participants were required to confirm their Puerto Rican heritage as indicated by having at least three Puerto Rican grandparents. Processes of informed consent, collection, processing, and storage of samples are published in detail elsewhere^19^. Briefly, we obtained de-identified stored peripheral blood samples from 122 healthy controls and 78 randomly selected cancer patients diagnosed with cancers other than melanoma (Supplemental Table 1) for isolation of DNA. Because *MC1R* is not known to be associated with cancers other than skin cancers, these cancer patients are considered representative of the general population. Information on diagnosis of non-melanoma skin cancers was unavailable from the PRBB. Ethics approval was obtained from the PHSU Institutional Review Board (IRB) Committee, and all research was performed in accordance with relevant regulations.

#### Community Participant Registry (CPR)

The CPR was a resource also developed as part of the PHSU-MCC partnership and comprised Hispanic residents in Tampa Bay and Puerto Rico who had provided consent to be contacted about cancer prevention and control research studies. Invitation packets that included an information statement, consent form, and questionnaire on demographics and pigmentation characteristics (Supplemental Table S2) were mailed to 176 eligible individuals; 39 (22%) returned the signed informed consent and questionnaire. These individuals were then asked to provide a saliva sample via a mailed Oragene^®^ DNA (DNA Genotek) kit. Mailed kits included detailed instructions to promote maximize yield and minimize contamination of collected saliva. Ethics approval was obtained from Chesapeake IRB with subsequent continuing renewal approval by Advarra, and all research was performed in accordance with relevant regulations.

#### Morsani Family Medicine clinics (MFMC), University of South Florida

To augment the number of Hispanics in our pilot study who lived in the Tampa Bay area, eligible patients (n = 105) at the MFMC were identified via clinical records and invited to participate at scheduled appointments. At the time of providing written informed consent, participants completed a questionnaire eliciting information on demographic variables and pigmentation characteristics (Supplemental Table S2) and provided a saliva sample using an Oragene^®^ DNA collection kit. Ethics approval was obtained from the Institutional Review Board of the University of South Florida, and all research was performed in accordance with relevant regulations.

#### 1000 Genomes Project

Genotype data at the *MC1R* locus were extracted from VCF files for the 104 Puerto Rican (PUR) participants available from the 1000 Genomes Project data portal^20^.

### Sample processing and genotyping

For DNA samples retrieved from the PRBB and obtained from participants recruited through the CPR and from the MFMC, we performed direct Sanger sequencing of the one exon coding region of *MC1R* to identify all existing variants^21^. We also genotyped 106 ancestry informative markers (AIM) that discriminate between Native American, African, and European ancestry. To maximize genetic information, SNPs with a large difference in allele frequency among ancestral populations were chosen. As well, representation across all 22 autosomal chromosomes was considered when selecting SNP markers. This AIM panel has been described previously^22^. AIM genotyping used a multiplex PCR coupled with single base extension methodology with alleles called using a Sequenom analyzer. Genotyping quality control for AIM was assessed using standard sample-level and SNP-level metrics. *MC1R* variants and genotype calls for the same 106 AIM were abstracted from 1000 Genomes genotyping data using VCFTools^23^.

#### MC1R variant categorization

We categorized participants into three groups based on the number and type of *MC1R* variant(s) carried using an algorithm similar to that described in Hernando *et al*.^24^ Participants in the low risk group did not carry any *MC1R* variant (consensus) or carried only variants that do not impact on receptor function, i.e. pseudoalleles, based on published functional analyses or as predicted from bioinformatical algorithms. Participants in the medium risk group carried only a single *MC1R* variant that results in partial loss of receptor function, i.e. “r” allele, based on published functional analyses or as predicted from bioinformatical algorithms. Participants in the high risk group carried either two “r” variants or carried at least one variant known to result in loss of receptor function based on published functional analyses or as predicted from bioinformatical algorithms.

#### Ancestry estimations

For each individual, global ancestry proportions were measured using the software Admixture v1.3 at a k = 3 and under a supervised model^25^. The parental reference populations were genotyped on the Affymetrix 100 K SNP chip, and included 42 Europeans (Coriell’s North American Caucasian panel), 37 West Africans (non-admixed Africans living in London, United Kingdom), and 30 Native Americans (15 Mayans and 15 Nahuas) and have been described previously^22^.

### Statistical analysis

*MC1R* minor allele frequencies were calculated. We compared *MC1R* risk categories by phenotypic skin cancer risk characteristics after dichotomizing phenotypic measures, and we used logistic regression models to determine odd ratios (OR) and corresponding 95% confidence interval (CI) with adjustment for gender. We compared *MC1R* risk categories by quartiles of European, African and Native American genetic ancestry and tested for differences in proportions using the Jonckheere-Terpstra test or the Cochran-Armitage trend tests. Quartiles of genetic ancestry were based on the overall participant sample (n = 315) that had successful ancestry genotyping. All analyses were conducted using SAS.

## Results

From the PRBB, 193 (97%) samples were successfully genotyped for *MC1R* (72 cases, 121 controls) and demographic information was obtained for 167 (87%) participants (49 cases, 118 controls). From the CPR, 32 (82%) of the responders completed the questionnaire on demographics and self-reported pigmentation characteristics and provided a saliva sample, and 30 (77%) samples were successfully genotyped for *MC1R*. Eighty-eight (84%) participants from the MFMC completed the questionnaire on demographics and self-reported pigmentation characteristics and provided a saliva sample; 79 (90%) samples were successfully genotyped for *MC1R*. Flow diagrams summarizing participant enrollment, biosample collection and genotyping, and availability for analyses are given in Supplemental Figure S1. Characteristics of these individuals with successful *MC1R* genotyping are summarized in Table 1.

**Table 1:** Individual demographic, pigmentation, and genetics characteristics according to sample population and overall.

	PRBB N = 193 N (%)	CPR N = 30 N (%)	MFMC N = 79 N (%)	1000 Genomes N = 104 N (%)	Overall N = 406 N (%)
**DEMOGRAPHIC**					
**Age**					
Years (mean ± SD)	50 ± 16.2	53 ± 11.0	49 ± 17.1	—	49.8 (16)
Missing	54 (30)	0	0	—	54 (17.9)
**Gender**					
Female	95 (49.2)	22 (73.3)	54 (68.4)	—	171 (56.6)
Male	49 (25.4)	8 (26.7)	25 (31.7)	—	82 (27.2)
Missing	49 (25.4)	0	0	—	49 (16.2)
**Education level**					
Less than or completed high school	41 (21.2)	8 (26.7)	23 (29.1)	—	72 (26.6)
Technical school or college	53 (27.5)	17 (56.7)	45 (57.0)	—	102 (37.6)
Graduate or professional school	36 (18.7)	4 (13.3)	11 (13.9)	—	33 (12.2)
Attended school outside of the USA	0	1 (3.3)	0	—	1 (0.4)
Missing	63 (32.6)	0	0	—	63 (23.2)
**Marital status**					
Single or never married	33 (17.1)	3 (10.0)	17 (21.5)	—	53 (17.0)
Married, civil union, or domestic partnership	43 (22.3)	16 (53.3)	48 (60.8)	—	117 (37.5)
Divorced, separated or widowed	5 (2.6)	11 (36.7)	14 (17.7)	—	30 (9.6)
Missing	112 (58.0)	0	0	—	112 (35.9)
**Ethnic subgroup**					
Puerto Rican only	193 (100.0)	20 (66.7)	38 (48.1)	104 (100)	355 (87.4)
Puerto Rican and other	0	0	6 (7.6)	0	6 (1.5)
Cuban only	0	0	7 (8.9)	0	7 (1.7)
Cuban and other	0	0	2 (2.5)	0	2 (0.5)
Dominican only	0	2 (6.7)	4 (5.1)	0	6 (1.5)
Mexican/Mexican American/Chicano only	0	1 (3.3)	4 (5.1)	0	5 (1.2)
Central or South American (other than Brazilian) only	0	6 (20.0)	16 (20.3)	0	22 (5.4)
Other only	0	0	2 (2.5)	0	2 (0.5)
Missing	0	1 (3.3)	0	0	1 (0.2)
**Race**					
White	89 (46.1)	29 (96.7)	62 (78.5)	—	180 (59.6)
Black or African American	11 (5.7)	1 (3.3)	10 (12.7)	—	22 (7.3)
Asian	0	0	0	—	0
Native Hawaiian or other Pacific Islander	0	0	0	—	0
American Indian or Alaska Native	1 (0.5)	0	0	—	1 (0.3)
Missing	92 (47.7)	0	7 (8.9)	—	99 (32.8)
**PIGMENTATION**					
**Eye color**					
Brown or black	—	25 (83.3)	76 (96.2)	—	101 (92.7)
Blue, gray, hazel, or green	—	5 (16.7)	3 (3.8)	—	8 (7.3)
Missing	—	0	0	—	0
**Freckling**					
None	—	15 (50.0)	58 (73.4)	—	73 (67.0)
Very few	—	10 (33.3)	14 (17.7)	—	24 (22.0)
Few or some	—	2 (6.7)	3 (3.8)	—	5 (4.6)
Many or very many	—	3 (10.0)	1 (1.3)	—	4 (3.7)
Missing	—	0	3 (3.8)	—	3 (2.8)
**Hair color**					
Red or blonde	—	6 (20.0)	1 (1.3)	—	7 (6.4)
Brown	—	13 (43.3)	39 (49.4)	—	52 (47.7)
Black	—	10 (33.3)	39 (49.4)	—	49 (45.0)
Missing	—	1 (3.3)	0	—	1 (0.9)
**Skin reaction to first strong summer sun**					
Tan and no sunburn	—	6 (20.0)	36 (45.6)	—	42 (38.5)
Mild sunburn followed by tanning	—	6 (20.0)	22 (27.8)	—	28 (25.7)
Sunburn without blister followed by some tanning	—	12 (40.0)	21 (26.6)	—	33 (30.3)
Severe sunburn and blister	—	5 (16.7)	0	—	5 (4.6)
Missing	—	1 (3.3)	0	—	1 (0.9)
**Skin reaction to long and repeated exposure to sun**					
Deeply tanned (very brown)	—	12 (40.0)	32 (40.5)	—	44 (40.4)
Moderately tanned	—	11 (36.7)	31 (39.2)	—	42 (38.5)
Mildly or occasionally tanned	—	4 (13.3)	14 (17.7)	—	18 (16.5)
No suntan but freckled	—	2 (6.7)	2 (2.5)	—	4 (3.7)
Missing	—	1 (3.3)	0	—	1 (0.9)
**GENETIC**					
***MC1R*** **risk category**					
Low	88 (45.6)	12 (40.0)	34 (43.0)	46 (44.2)	180 (44.3)
Medium	58 (30.1)	12 (40.0)	30 (38.0)	37 (35.6)	137 (33.7)
High	47 (24.4)	6 (20.0)	15 (19.0)	21 (20.2)	89 (21.9)
**Genetic ancestry**					
Ancestry informative marker genotyping completed	181 (93.8)	0 (0)	30 (38.0)	104 (100)	315 (77.6)
African (mean percent, standard deviation)	18% (10)	—	22% (20)	15% (12)	17% (12)
European (mean percent, standard deviation)	68% (12)	—	59% (21)	72% (14)	68% (14)
Native American (mean percent, standard deviation)	14% (7)	—	19% (16)	13% (7)	14% (8)

### *MC1R* genotype

Including the 104 Puerto Rican participants from the 1000 Genomes Project, a total of 406 individuals had an available *MC1R* genotype. Of these individuals, 137 (34%) were in the medium risk group and 89 (22%) were in the high risk group (Table 1). By definition, all individuals in the medium risk group were heterozygous carriers of an r allele. Among the 89 participants in the high risk group, two (2.3%) were heterozygous compound carriers of two R alleles, 20 (22.5%) carried one R and one r allele, 22 (24.7%) carried two r alleles (six in a homozygous state), and 45 (50.6%) carried one R allele. Table 2 summarizes the minor allele frequencies in individuals of sole Puerto Rican heritage, who comprise the majority (87%, n = 355) of participants in this study. Except for the D84E variant, each of the other nine most well-described risk variants (V60L, V92M, R142H, R151C, I155T, R160W, R163Q, D294H) was observed. Twenty-six observances of 12 rare non-synonymous or insertion/deletion variants were detected in our study participants, but none were novel.

**Table 2:** Minor allele frequencies of *MC1R* variants observed among subjects with sole Puerto Rican heritage obtained or recruited from the Puerto Rico Biobank, Community Participant Registry, Morsani Family Medicine clinics, and the 1000 Genomes Project, and overall.

*MC1R* variant	rs Number	Pilot study (N^a^ = 251)	1000 Genomes Project (N^a^ = 104)	Overall (N^a^ = 355)
		Frequency (%)	Frequency (%)	Frequency (%)
**Non-synonymous**				
F45L	rs767905960	0.4	0	0.3
S47I	rs371156858	0.2	0	0.1
V60L	rs1805005	9.2	11.5	9.9
A64T	rs368501338	0.2	0	0.1
D84E	rs1805006	0	0	0
D84N	rs538777064	0	0.5	0.1
V92M	rs2228479	3.8	2.9	3.5
A111V	rs201489928	1.0	0	0.7
R142C	rs752927306	0.2	0	0.1
R142H	rs11547464	0.2	0.5	0.3
R151C	rs1805007	2.4	2.4	2.4
Y152X	rs201326893	0	0.5	0.1
I155T	rs1110400	1.6	1.9	1.7
R160W	rs1805008	0.4	0.5	0.4
R163Q	rs885479	10.8	9.6	10.4
F196L	rs3212366	1.2	0.5	1.0
D294H	rs1805009	2.4	1.4	2.1
C315R	rs761041641	0.4	0	0.3
**Insertion/deletion**		0.6	0.5	0.6
g.86_87insA	rs796296176	0	0.5	0.1
g.158_160delTGG	rs779655156	0.2	0	0.1
g.537_538insC	rs555179612	0.4	0	0.3
**Synonymous**		5.4	0	3.8
L106L	rs3212364	0	1.0	0.3
A111A	rs368745976	0.2	0.5	0.3
I168I	rs34612847	0.6	0	0.4
Y298Y	rs143395134	0	0.5	0.1
F300F	rs3212367	1.6	1.0	1.4
T314T	rs2228478	3.0	13.5	6.1

^a^N = number of participants; number of chromosomes for calculation of minor allele frequencies is double this number.

### *MC1R* genotype and pigmentation characteristics

Among CPR and MFMC participants who completed a questionnaire capturing phenotypic information, most reported having darker phenotypic characteristics (Table 1). Associations among *MC1R* risk categories and pigmentation characteristics are given in Table 3. We noted a significant trend (p = 0.0004) across *MC1R* risk categories with tendency to burn: Hispanic individuals in the medium (OR: 3.4, 95% CI: 1.2–9.2; p = 0.017) and high (OR: 8.4, 95% CI: 2.5–28; p = 0.0005) *MC1R* risk categories were more likely to report skin that burned (including sunburn without blistering and severe burning with blistering) compared to those in the low *MC1R* risk category. A similar, but weaker, trend (p = 0.025) was noted with tendency to tan: Hispanic individuals in the medium (OR: 2.0, 95% CI: 0.61–6.7; p = 0.25) and high (OR: 4.4, 95% CI: 1.2–16; p = 0.025) *MC1R* risk categories were more likely to report skin that only mildly tanned at best compared to those in the low *MC1R* risk category. Only the trend for burning remained significant after Bonferroni correction for multiple comparisons. Although individuals in the medium (OR: 2.0, 95% CI: 0.75–5.5) and high (OR: 2.4, 95% CI: 0.76–7.8) *MC1R* risk categories were more likely to freckle (including very few, few, some, many, or very many) compared to individuals in the low risk category, associations were not statistically significant. We did not find an association between *MC1R* risk categories and eye or hair color, in part because of the limited proportion (6–7%) of individuals reporting light eye color (including blue, grey, green, or hazel) or red or blonde hair color.

**Table 3:** Association of *MC1R* variants and phenotypic characteristics of participants recruited from the Community Participant Registry or Morsani Family Medicine clinics.

Phenotypic factors	*MC1R* risk category^a^					*P*	OR^c^ (95% CI)	*P*	*P*_trend_
	Low (n=46) *N (%)*	Medium (n=42) *N (%)*	High (n=21) *N (%)*	OR^b^ (95% CI)					
**Eye color**									
Brown or black	43 (93.5)	40 (95.2)	18 (85.7)	1.0			1.0		
Blue, gray, hazel or green	3 (6.5)	2 (4.8)	3 (14.3)	0.77 (0.12-4.9)		0.78	2.7 (0.47-15.1)	0.27	0.32
**Hair color**									
Black	21 (46.7)	18 (42.9)	10 (47.6)	1.0			1.0		
Brown	21 (46.7)	21 (50.0)	10 (47.6)						
Red or blonde	3 (6.7)	3 (7.1)	1 (4.8)	1.2 (0.22-6.3)		0.85	0.80 (0.075-8.4)	0.85	0.92
**Skin reaction to first strong summer sun (burnability)**									
Tan and no sunburn	25 (55.6)	14 (33.3)	3 (14.3)	1.0			1.0		
Mild sunburn followed by some tanning	12 (26.7)	11 (26.2)	5 (23.8)						
Sunburn without blister followed by some tanning	7 (15.6)	15 (35.7)	11 (52.4)	3.4 (1.2-9.2)		0.017	8.4 (2.5-28)	0.0005	0.0004
Severe sunburn and blister	1 (2.2)	2 (4.8)	2 (9.5)						
**Skin reaction to long and repeated exposure to sun (tanning ability)**									
Deeply tanned (very brown)	23 (51.1)	16 (38.1)	5 (23.8)	1.0			1.0		
Moderately tanned	17 (37.8)	17 (40.5)	8 (38.1)						
Mildly or occasionally tanned	4 (8.9)	8 (19.1)	6 (28.6)	2.0 (0.61-6.7)		0.25	4.4 (1.2-16)	0.025	0.025
No suntan but freckled	1 (2.2)	1 (2.4)	2 (9.5)						
**Freckling**									
None	35 (79.6)	26 (63.4)	12 (57.1)	1.0			1.0		
Very few	8 (18.2)	11 (26.8)	5 (23.8)	2.0 (0.75-5.5)		0.16	2.4 (0.76-7.8)	0.14	0.11
Few or some	1 (2.3)	2 (4.9)	2 (9.5)						
Many or very many	0 (0)	2 (4.9)	2 (9.5)						

^a^*MC1R* risk categories are defined as low (carriage of no variants or only variants without demonstrated or predicted impact upon receptor function), medium (sole carriage of a single variant with known demonstration or predicted partial loss of receptor function), and high (carriage of two medium risk variants or carriage of variants with known demonstration or predicted loss of function of receptor function).

^b^OR for carriage of medium vs. low risk *MC1R* variants; OR adjusted for gender.

^c^OR for carriage of high vs. low risk *MC1R* variants; OR adjusted for gender.

### Genetic ancestry

Genotyping of AIM was completed on 181 samples from the PRBB, 30 individuals recruited from the MFMC, and was available on 104 Puerto Rican individuals from the 1000 Genomes Project. Mean genetic ancestry proportions for these 315 individuals are listed in Table 1, and Supplemental Table S3 displays mean genetic ancestries according to Hispanic heritages. Table 4 displays the proportions of participants in the low, medium, and high *MC1R* risk categories according to quartiles of genetic ancestry among individuals of sole Puerto Rican heritage. Within European, African, and Native American genetic ancestries, the proportions of Hispanic participants carrying low, medium, or high *MC1R* risk variants were similar across genetic quartiles, and all statistical tests were not significant (p > 0.05).

**Table 4:** Quartiles of genetic ancestry by *MC1R* genetic risk group among subjects with sole Puerto Rican heritage obtained or recruited from the PRBB, CPR, Family Medicine clinics, and the 1000 Genomes Project.

Quartiles of genetic ancestry^a^	*MC1R* risk category		
	Low (N = 131)	Medium (N = 97)	High (N = 69)
	N (%)	N (%)	N (%)
**European**			
<59.9%	30 (22.9)	21 (21.7)	17 (24.6)
≥59.9% and <70.2%	35 (26.7)	26 (26.8)	17 (24.6)
≥70.2% and <78.1%	35 (26.7)	32 (33.0)	11 (15.9)
≥78.1%	31 (23.7)	18 (18.6)	24 (34.8)
P-value^b^	0.65		
P-value_(low/medium vs. high)_^c^	0.47		
P-value_(low vs. medium/high)_^d^	0.85		
**African**			
<9.7%	35 (26.7)	20 (20.6)	19 (27.5)
≥9.7% and <15.4%	28 (21.4)	25 (25.8)	18 (26.1)
≥15.4% and <22.0%	34 (26.0)	28 (28.9)	17 (24.6)
≥22.0%	34 (26.0)	24 (24.7)	15 (21.7)
P-value^b^	0.67		
P-value_(low/medium vs. high)_^c^	0.38		
P-value_(low vs. medium/high)_^d^	0.97		
**Native American**			
<9.0%	30 (22.9)	25 (25.8)	16 (23.2)
≥9.0% and <13.8%	40 (30.5)	21 (21.7)	17 (24.6)
≥13.8% and <19.0%	36 (27.5)	29 (29.9)	14 (20.3)
≥19.0%	25 (19.1)	22 (22.7)	22 (31.9)
P-value^b^	0.28		
P-value_(low/medium vs. high)_^c^	0.31		
P-value_(low vs. medium/high)_^d^	0.37		

^a^Quartile cutpoints are based on the distribution of genetic ancestry observed the overall participant sample with successful ancestry genotyping (n = 315).

^b^P-value is from the Jonckheere-Terpstra test comparing the proportion of participants in *MC1R* risk categories across quartiles of genetic ancestry.

^c^P-value is from the Cochran-Armitage test for trend comparing the proportion of participants in a combined low and medium *MC1R* risk category to that in the high *MC1R* risk category across quartiles of genetic ancestry.

^d^P-value is from the Cochran-Armitage test for trend comparing the proportion of participants in the low *MC1R* risk category to that in a combined medium and high *MC1R* risk category across quartiles of genetic ancestry.

## Discussion

This pilot study on the prevalence of *MC1R* variants in a Hispanic population in Tampa and Puerto Rico found that 56% of participants carried a *MC1R* allele(s) that placed them at elevated risk for skin cancer, the vast majority of which have been shown to increase the odds of melanoma, SCC, or BCC by at least 80%^6,8^. Our analyses demonstrate that *MC1R* variants are associated with some pigmentation characteristics in this overwhelmingly Puerto Rican sample, consistent with observations seen in European populations^26^. Among individuals reporting only Puerto Rican heritage, the proportion of participants categorized at elevated *MC1R* risk was comparable across quartiles of European ancestry, across quartiles of African ancestry, and across quartiles Native American genetic ancestry, indicating that those with greater African or Native American genetic ancestry carried *MC1R* risk alleles. Although our findings are limited by the overall and sub-group sample sizes and self-reported pigmentation characteristics, these results contribute novel evidence on the *MC1R* gene in an underserved population, which will inform future research studies.

Currently, our understanding of *MC1R* variants and their association with skin cancer risk is based on studies predominately in non-Hispanic whites. However, some international studies in general population and melanoma family settings have demonstrated a range in the prevalence and variation of *MC1R* variants according to geographic location^27,28^. Studies conducted in Spanish populations have found approximately 50–70% of individuals carry at least one risk variant^24,29,30^, which is comparable to the combined prevalence of medium and high risk alleles in our study. One study compared the association between melanoma and *MC1R* variants in German and Spanish populations, and the authors found significant differences in the frequency of, and risk attributable to, *MC1R* variants in the two populations^31^.

In our study, the overall proportions of genetic ancestry were consistent with those from published data, which demonstrate that Hispanic and Latino populations have high admixture of predominately European, African, and Native American ancestry^32^. We observed minimal variation in *MC1R* risk according to genetic ancestry among Hispanics of Puerto Rican heritage. Our findings suggest that *MC1R* variants are relevant to a diverse population and that even among populations with less European and stronger African or Native American genetic ancestry there may be carriers of alleles conferring elevated risk. Furthermore, these data reinforce the need for future research on the varied functional impact of *MC1R* variants according to population subgroups that are genetically and geographically diverse^31^.

Several studies among non-Hispanics have shown that genetic variation at *MC1R* is associated with melanoma risk independent of traditional phenotypic characteristics (e.g. hair and skin color) and that *MC1R* may even confer higher risk among individuals with a darker phenotype than among those with a lighter phenotype^21,27,28^. However, to the best of our knowledge, studies of associations between *MC1R* and skin cancer risk in Hispanic populations are lacking. Some studies have reported on the prevalence of some *MC1R* risk variants in Hispanic and Latinx sub-groups such as Mexican-American, Uruguayan and Brazilian, but most have either focused on populations at elevated risk of melanoma due to personal/family disease history^33,34^ or they were conducted in the context of other diseases, such as depression^35,36^. One recent study set in New Mexico reported carriage of a medium or high risk *MC1R* variant in 63% of enrolled Hispanics^37^. Some studies have examined associations between *MC1R* variation and genetic ancestry^38,39^, but there appears to be limited research that also incorporates pigmentation characteristics in Hispanic populations. Our findings contribute novel evidence on the association between *MC1R* variation and pigmentation characteristics in Hispanics who were not selected to participate on the basis of their personal or family history of melanoma (or non-melanoma skin cancers).

The belief that darker skin pigmentation is infallibly protective against skin cancer can serve as a barrier to Hispanics undertaking, and receiving education in, prevention and early detection behaviors^16^. As expected, the majority of participants in our study who completed the questionnaire reported darker phenotypic characteristics. Among those who reported moderate to strong tanning ability, 53% carried either medium or high *MC1R* risk variants, and this group particularly stands to benefit from receiving information on their *MC1R* genotype as it may change their perceived skin cancer risk (from lower to higher). High uptake and interest in *MC1R* testing among Hispanics has previously been demonstrated^18^, and the impact of receiving such genetics-based skin cancer risk information on behavioral, psycho-social and ethical outcomes is being investigated in ongoing trials in Hispanic and broader population contexts^40,41^. Additionally, our findings indicate a need for further research on the pathways and attributable risk of *MC1R* variants in individuals with sun resistant phenotypes.

This pilot study demonstrated that risk information based on *MC1R* genetic variants may be relevant to a diverse, Hispanic population and could inform skin cancer risk assessment, prevention and early detection recommendations in this setting. Our findings further highlight the need to ensure that prevention and early detection recommendations are inclusive of populations with low risk phenotypic characteristics alongside general population strategies, and the need for research on the pathways and risk of *MC1R* variants in groups with diverse genetic ancestry and phenotypic characteristics.

## Supplementary information

Supplementary information.

## Data Availability

The datasets generated during and/or analyzed during the current study are available from the corresponding author on reasonable request.
