# Supplementary material for: MC1R variants and associations with pigmentation characteristics and genetic ancestry in a Hispanic, predominately Puerto Rican, population
Source: Sci Rep. 2020 Apr 29;10:7303. doi: 10.1038/s41598-020-64019-y (PMC7190662; doi:10.1038/s41598-020-64019-y)
Supplement: Supplementary file 1 — Supplementary information. [file 41598_2020_64019_MOESM1_ESM.pdf]

Supplemental information

***MC1R* variants and associations with pigmentation characteristics and genetic ancestry in a Hispanic, predominately Puerto Rican, population**

Amelia K. Smit, Marielys Collazo-Roman, Susan T. Vadaparampil, Stella Valavanis, Jocelyn Del Rio, Brenda Soto, Idhaliz Flores, Julie Dutil, Peter A. Kanetsky

Supplementary Table S1. Cancer types in 78 cancer patients from the Puerto Rico Biobank.

Cancer type	N (%)
Breast	6 (8%)
Endometrial	11 (14%)
Esophagus	1 (1%)
Kidney	3 (4%)
Leukemia	3 (4%)
Lung	6 (8%)
Lymphoma	1 (1%)
Prostate	36 (46%)
Rectum	3 (4%)
Sigmoid Colon	1 (1%)
Thyroid	7 (9%)

Supplementary Table S2. Questionnaire completed by participants recruited from the Community Participant Registry or Morsani Family Medicine clinics.

What is your sex?

- Male
- Female

In what year were you born?

What is your gender?

- Female
- Male

What is the highest grade or level of school you have completed?

- Less or completed high school
- Technical school or College
- Graduate or Professional School
- Attended school outside of the USA

Which of the following describes your current relationship status?

- Single or never married
- Married, civil union or domestic partnership
- Divorced, separated or widow

Do you consider yourself to be Hispanic or Latino (of Spanish origin)?

- Yes
- No

Which group or groups best represent your Hispanic or Latino heritage? (Check all that apply)

- Puerto Rican
- Cuban
- Dominican (Republic)
- Mexican, Mexican American, Chicano
- Central of South American, other than Brazilian
- Other, specify ____________________

Which of the following best describes you? ***Please select all that apply.***

- White
- Black or African American
- Asian
- Native Hawaiian or other Pacific Islander
- American Indian or Alaska Native
- Other
- Multiple

Which color best describes your natural hair color at age 18?

- Red
- Fair or Blonde
- Brown
- Black

Which color best describes the color of your eyes?

- Blue
- Brown or Black
- Any other color

How many freckles you would have on your face at the end of the summer?

None
Very few
Few
Some
Many
Very Many

Which statement best describes what would happen if your skin were exposed to bright sunlight for the first time in summer for one hour in the middle of the day without any protection?

Get a severe sunburn with blistering?
Have a painful sunburn for a few days followed by peeling?
Get mildly sunburned followed by some tanning?
Go brown without any sunburn?

Which of the following best describes what would happen to your skin if it were repeatedly exposed to bright sunlight in summer without any protection?

Go very brown and deeply tanned
Get moderately tanned
Get mildly or occasionally tanned
Get no suntan at all or only get freckled

Have you ever had a full-body skin examination where all parts of your body were carefully examined for unusual looking moles?

Yes
No

***[If yes]* Within the past year, have you had a full-body skin examination where all parts of your body were carefully examined for unusual looking moles?**

Yes
No

***[If yes]* Who completed your most recent full-body skin examination?**

Myself
My spouse or partner
A trained health professional (Physician or nurse)

Have you ever been diagnosed with melanoma?

Yes
No

Has anyone in your family ever been diagnosed with melanoma?

Yes
No
Don’t know

Has anyone in your family ever been diagnosed with skin cancer that was not melanoma?

Yes
No
Don’t know

Has anyone in your family ever been diagnosed with any other type of (non-skin) cancer?

Yes
No
Don’t know

Supplementary Table S3. Genetic ancestry by groups defined by degree of reported Puerto Rican heritage.

Genetic ancestry	Puerto Rican only (N=297)	Combined Puerto Rican and other (N=4)	Other Hispanic heritage only (N=14)
	Mean % (SD; range)*	*Mean % (SD; range)*	*Mean % (SD; range)*
**European**	70% (13; 13-100)	57% (22; 34-87)	59% (25; 28-95))
**African**	17% (11; 0-75)	25% (27; 10-66)	21% (24; 0-67)
**Native American**	14% (7; 0-48)	18% (19; 0-36)	20% (18; 1-61)

* Sum of mean genetic ancestries >100% due to rounding.

Supplementary Figure S1 (following page). Flow diagrams of participant enrollment, biosample collection and genotyping, and availability for analyses for the Community Participant Registry, Morsani Family Medicine clinics, Puerto Rico Biobank, and the 1000 Genomes project.

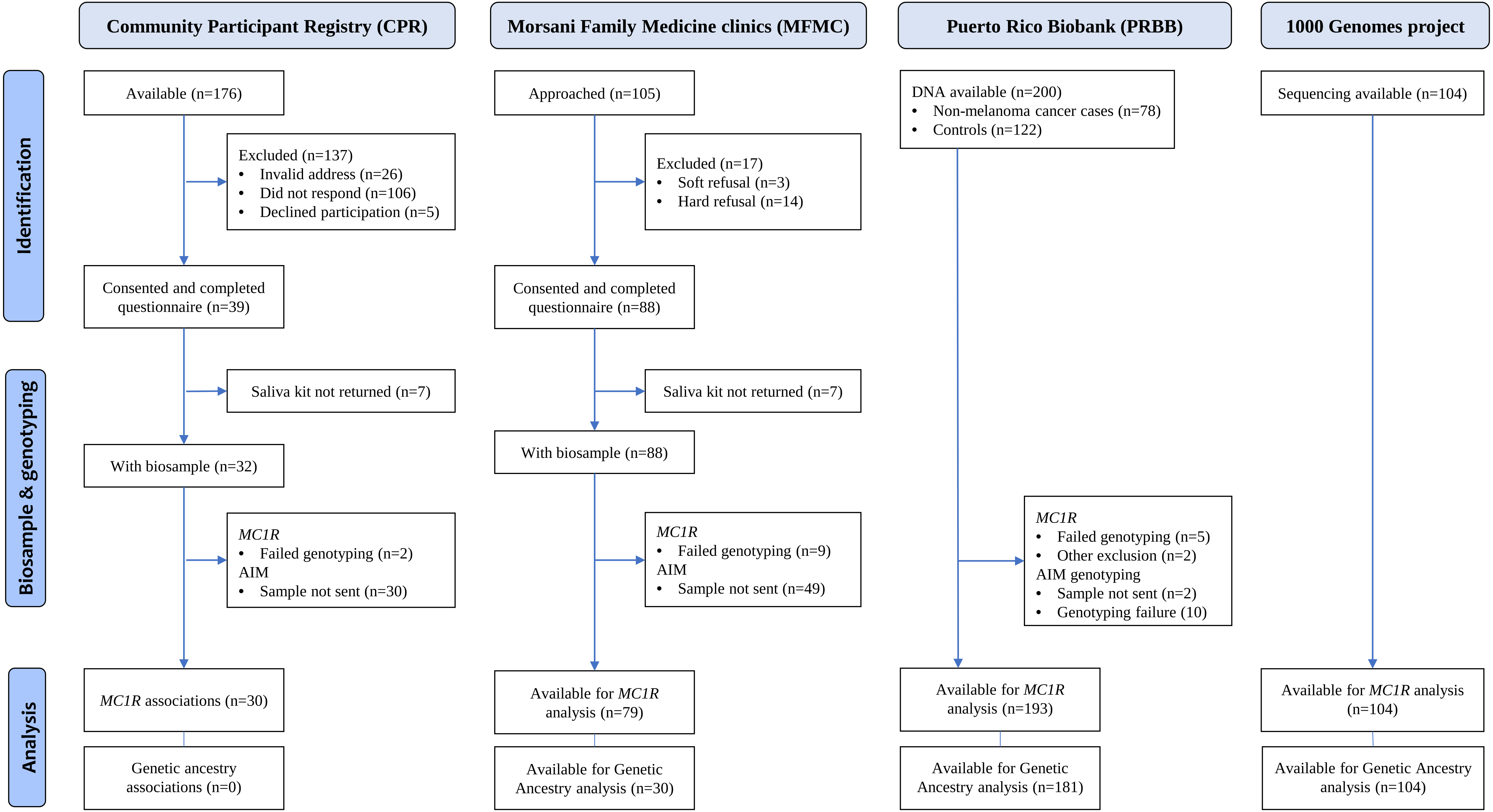
Community Participant Registry (CPR)
Morsani Family Medicine clinics (MFMC)
Puerto Rico Biobank (PRBB)
1000 Genomes project
Identification
Biosample & genotyping
Analysis
Available (n=176)
Excluded (n=137)
• Invalid address (n=26)
• Did not respond (n=106)
• Declined participation (n=5)
Consented and completed questionnaire (n=39)
Saliva kit not returned (n=7)
With biosample (n=32)
MC1R
• Failed genotyping (n=2)
AIM
• Sample not sent (n=30)
MC1R associations (n=30)
Genetic ancestry associations (n=0)
Approached (n=105)
Excluded (n=17)
• Soft refusal (n=3)
• Hard refusal (n=14)
Consented and completed questionnaire (n=88)
Saliva kit not returned (n=7)
With biosample (n=88)
MC1R
• Failed genotyping (n=9)
AIM
• Sample not sent (n=49)
Available for MC1R analysis (n=79)
Available for Genetic Ancestry analysis (n=30)
DNA available (n=200)
• Non-melanoma cancer cases (n=78)
• Controls (n=122)
MC1R
• Failed genotyping (n=5)
• Other exclusion (n=2)
AIM genotyping
• Sample not sent (n=2)
• Genotyping failure (10)
Available for MC1R analysis (n=193)
Available for Genetic Ancestry analysis (n=181)
Sequencing available (n=104)
Available for MC1R analysis (n=104)
Available for Genetic Ancestry analysis (n=104)
